# Race-Level Reporting of Incidents during Two Seasons (2015/16 to 2016/17) of Harness Racing in New Zealand

**DOI:** 10.3390/ani12040433

**Published:** 2022-02-11

**Authors:** Michaela J. Gibson, Fernando J. Roca Fraga, Charlotte F. Bolwell, Erica K. Gee, Chris W. Rogers

**Affiliations:** School of Veterinary Sciences, Massey University, Palmerston North 4442, New Zealand; m.gibson@massey.ac.nz (M.J.G.); f.roca@massey.ac.nz (F.J.R.F.); c.bolwell@massey.ac.nz (C.F.B.); e.k.gee@massey.ac.nz (E.K.G.)

**Keywords:** harness racing, incident, non-incident, steward, stipendiary report, injury, poor performance, equine welfare

## Abstract

**Simple Summary:**

The objective of this study was to describe the incident reporting of harness racing in New Zealand. Retrospective stipendiary stewards’ reports of race day events during the 2015/16 to 2016/17 racing season were examined to describe the reasons and outcomes for race day veterinary examinations of Standardbred horses in New Zealand. The primary reason for examination of horses after a race was due to poor performance. Poor performance was considered if a horse’s performance in the race was lower than its previous race, or lower than expected as reflected by the odds at the tote (reflecting the amount of money placed/gambled on the horse via the official betting agency). The lack of fatalities and injuries reported indicates a low risk profile in harness racing and highlights the stewards’ role in maintaining racing integrity and animal welfare.

**Abstract:**

The objective of this study was to describe the incident and non-incident reporting of harness racing in New Zealand, the primary injury and reporting outcomes, and to examine horse- and race-level variables associated with the odds of these outcomes. Retrospective stipendiary stewards’ reports of race day events during the 2015/16 to 2016/17 racing seasons were examined. The number of incident and non-incident events and binomial exact 95% confidence intervals (CI) were calculated per 1000 horse starts. Most reports were for non-incidents and an examination was requested for poor performance (11.06 per 1000 starts (95% CI = 10.23–11.89). Races with more than eight participants were 1.9 (95% CI = 1.13–3.4) times more likely to have an incident than races with eight or less participants. The low incidence of significant injuries such as fractures (0.13 per 1000 starts (95% CI = 0.03–0.23) reflects the lower risk of injury in harness racing compared to Thoroughbred racing. The high incidence of poor performance reports highlights the steward’s role in maintaining animal welfare to a high standard.

## 1. Introduction

While smaller in participation numbers and betting turnover than Thoroughbred racing, harness racing represents a significant racing industry and has received considerably less attention in the scientific literature than Thoroughbred racing. Harness racing is conducted using Standardbred horses that race at either a trotting or pacing speed whilst drawing a two-wheeled cart called a sulky [1]. Within New Zealand, the structure of the harness racing industry and Standardbred breeding industry have been described [2,3]. There are limited data on the training of standardbreds internationally, [4] or from within New Zealand [5], and little information on injuries and injuries associated with racing events [6]. This trend is also observed in the international literature with limited data on racing related injuries in standardbred harness racing [4,7].

Within the regulatory process of racing, most jurisdictions utilize a process of stipendiary steward reporting and veterinary reports. These reports are routinely collected during race meetings and are published as part of the transparency of racing integrity [7]. Reports are identified as either a non-incident or an incident report. Non-incident reports occur when there has been no identifiable “event” during a race, and routine screening of horses is required as part of the ongoing regulatory and integrity process. The screening requests often focus on a horse or horses that may not have performed up to expectations, or if a horse’s health is questioned (e.g., suspected epistaxis). An incident report is the result of an “event” before or during a race (such as a horse collision, stumble, fall or the horse “breaks its stride”) that requires a horse to be examined. Both reports involve an assessment from the designated veterinarian(s) on duty for the race meeting.

Stipendiary stewards’ reports provide an indication of the robustness of the regulatory process and the screening of horses during a race meeting [7]. When combined with race data, this information provides the opportunity to describe the incidence of injuries (from mild lacerations to catastrophic injury) and the odds of these events, or outcomes, with different horse and environment level variables. The collection of such data is important for the monitoring of industry practice and to optimize horse welfare. Such data permit evidence-based changes to be made to management and the structure of racing to meet the industry’s duty of care to the horse racing in it. External to these is the obligation of the industry to minimize injury and loss to meet its social license to operate [8].

To date, most of the publications describing race day events from stipendiary stewards or race day veterinary events have examined the Thoroughbred racing industry. International and domestic data have suggested that potential risk factors for race day injury in Thoroughbred racing include track surface, track condition, race distance, race class, age of horse, training intensity, and number of starters [9,10,11]. Within harness racing, Physick-Sheard, Avison, and Sears [4] identified that sex, age, track class, performance history, and workload affect the likelihood of horse mortality on race day. However, they did not examine non-fatal injuries. Australian studies of harness racing steward reports have been carried out to look at the incidence of injuries, mortality, and reasons for poor performance providing a benchmark to assess ongoing improvements in welfare [7]. Lameness was the most common finding in post-race examinations with 2.1 cases per 1000 starts, followed by poor performance/heat stress with 2.04 cases per 1000 starts. 

To monitor changes in New Zealand harness racing welfare over time, the prevalence of the current incident and non-incident reporting needs to be measured. Therefore, the objective of this study was to describe the incident and non-incident reporting during the 2015/2016 and 2016/2017 harness racing seasons in New Zealand, the primary injury and reporting outcomes, and to examine horse- and race-level variables associated with the rate of the reporting of these outcomes.

## 2. Materials and Methods

Data were obtained of all race starts during the 2015/16–2016/17 racing season as an excel spreadsheet from Harness Racing New Zealand, the official registration body for Harness racing in New Zealand. An Excel spreadsheet of all stipendiary steward reports for the same racing seasons were obtained from the Racing Integrity Unity, the official racing compliance organization for all three racing codes within New Zealand. Each stipendiary steward report recorded the date, racecourse, race number, horse name, and other information relevant to the incident reported such as the reason for the requested report and the findings of the veterinary examination. To obtain information about racing surface (grass and all-weather) and condition (fast, good, dead, slow, heavy, easy, and slushy), race type and pace, horse age, and other relevant track and horse information, stipendiary steward reports were cross-referenced with the official race start records and results from the same period.

### Statistical Analysis

Data were exported to RStudio (version 3.5. 1, 2018; R Foundation for Statistical Computing, Vienna, Austria) for manipulation and analysis. After merging of the two datasets, data were cross validated using the horse’s official race name as the unique identifier. Apparent errors or inconsistencies in data (i.e., misspelt horses names) were then checked manually against the official formal transcript of the relevant stipendiary stewards’ report hosted and archived on the Harness Racing New Zealand website. Horses that did not race, or steward reports that were miscategorized (thoroughbred reports), were removed from the dataset. Six records were excluded due to mis-entered values with data that prevented linkage between the two databases. There were 87 reports that recorded an event occurring prior to the beginning of the race meeting that resulted in horses being withdrawn from the race meeting. Using the official descriptors provided within the datasheets, reports were coded for analysis as incident and non-incident reports. Non-incident reports occur when there has been no identifiable “event” during a race, and routine screening of horses is required as part of the ongoing regulatory and integrity process. An incident report is the result of an “event” before or during a race (such as a horse collision, trip or fall) that requires a horse to be examined. Both reports involve an assessment from the designated veterinarian(s) on duty for the race meeting. Multiple phrases and spellings (abbreviations) were used to describe reasons for a horse examination and the outcome findings. These were manually coded and collapsed down into 14 categories, these being Arrhythmia, Cardiac failure, Laceration/abrasion, Lame, Musculoskeletal injury (Fracture), Other musculoskeletal (MS) issues, Unknown musculoskeletal pain, no observable abnormalities detected (NOAD), Poor recovery, Respiratory issues, Previous injury, Bleeders (epistaxis), Unknown and Miscellaneous.

Track conditions on grass surfaces were defined as fast, good, dead, slow and heavy. Track conditions on all weather surfaces were defined as fast, good, easy or slushy.

Statistical analysis was conducted using SAS version 9.4 (SAS Institute Inc., Cary, NC, USA). Descriptive statistics (mean, median and proportion) were used to describe the data at a season, track, and race level. Incidence rates are described as the number of events per 1000 starts with their 95% confidence intervals (Parkin [9])

Data were tested for normality and if non-parametric, differences between groups were tested using the Kruskall–Wallis test and reported as median and interquartile range (IQR). The distribution of stewards’ reports, and the underlining demographics of the industry and race conditions were initially examined using the Chi-Squared test. Univariable logistic regression was used to screen variables for association with both incident and non-incident reporting. 

## 3. Results

### 3.1. Harness Racing Data

During the 2015/16–2016/17 seasons, 4211 horses had a race start, over half of the racing population were geldings (53%), followed by mare and fillies (43%), then stallions and colts (4%). Of these 4211 horses, 3034 (72%) had at least one start in the 2015/16 season and 2923 (69%) had at least one start in the 2016/17 season. These horses had 54,702 racing starts, of which 27,819 (50.9%) were during the 2015–2016 season and 26,883 (49.1%) during the 2016–2017 season. Most of starts were for horses in pacing races (72% of starters and 73% of races). The racing took place at 40 racecourses, 22 of which were grass tracks with the remainder being all weather tracks. Most starts (*n* = 44,280 (80.9%)) were on all-weather surfaces and 10,422 (19.1%) on grass. Of the 44,280 starts on all-weather surfaces, the majority of the racing surface conditions were described as “fast” (*n* = 32,678 (73.8%)), the remaining being described as on a “good” (*n* = 6574 (14.8%)), “easy” (*n* = 2375 (5.4%)) or “slushy” (*n* = 2653 (6%)). Of the 10,422 starts on grass, surface conditions were described as “fast” (1255 starts or 12%), “good” (6956 starts or 67%), “dead” (1367 starts 13.1%), “slow” (712 starts or 6.8%) and “heavy” (132 starts or 1.3%). There was a median race distance of 2200 m (IQR 2000–2600) with 97% (53088/54702) of starts being over 1609 m (1 m). The median mile rate was 2.04 min (IQR 2.0–2.09) for pacing races and 2.14 min (IQR 2.09–2.20) for trotting races. Most races were at racetracks located in the southern region (South Island) (65.6% 3370/5139) followed by the northern region (26.2%, 1346/5139) (Top half of the North Island) with few races in the central region (Bottom half of the North Island) (8.2%, 423/5139). 

There was a median of 11 starters per race (IQR 9–13). The horses participated in a median of 7 (IQR 3–10) races per season with no difference between the 2015/16 and 2016/17 season for number of starts. Younger horses participated in fewer starts per season, 2-year-olds in a median of 3 (IQR 2–5) starts per year, 3-year-old horses in 6 (IQR 3–10) starts and 4+yo horses in 9 (IQR 4–15) starts per season (*p* < 0.001). A detailed description of the distribution of age and sex is presented in Table 1. 

### 3.2. Stewards Reports

The 1001 records that were included in this study were coded into two categories: (a) screening due to major events occurring before (from the start of the race meeting), during and after the race as “incident reports” (*n* = 131) and (b) screening due to poor performance or other non-incident concerns, often as part of routine racing integrity screening, coded as “non-incident reports” (*n* = 870). Most of the stipendiary stewards’ reports described events relating to races (96.3%, 964/1001) rather than those occurring either before the race (2.7%,27/1001), post-race (0.80% 8/1001) or unknown/not described in the report (0.20%, 2/1001).

There were only nine (0.16 times per 1000 races, 95% CI = −0.08–0.41) musculoskeletal fractures were reported in the stipendiary stewards’ reports for both seasons. 

#### 3.2.1. Incident Reports

An incident report occurred every 2.4 per 1000 starts (95% CI = 2.0–2.8). The distribution of incident reports across race types reflected the underlying racing population with 77.4% (102/131) associated with pacing races and the remaining 22.6% (29/131) with trotting races (*p* < 0.05). There was no association of track surface with the frequency of incident reports (*p* = 0.674). Track conditions were reported as fast for 60% of incidents followed by good (32.1%), easy (2.3%), slushy (3.8%) and slow (0.8%) and dead (0.8%). There were no incidents on heavy tracks. There was no association of track condition with the rate of incident reporting (*p* = 0.353). There was a positive association of field size with incident reports such that an incident was 1.9 [95% CI = 1.13–3.4, (*p* < 0.001)] times more likely to occur in a race with 9 or more participants than in a race with 8 or less participants. Incident reports reflected the average number of participants per race on each surface. 

There were a variety of reasons recorded for the requesting of an incident report with the largest category being due to a horse falling (0.79/1000 starts [95% CI = 0.66–0.91]; 33.0%), primarily during the race (64.3% of falling horses). The major clinical finding for falling horses was integument lacerations and/or abrasions (52.4%) with the remaining 47.6% having no clinical finding. Only 8.8% of horses reported in incident reports (0.13 per 1000 starts, 95% CI = 0.03–0.23) were coded as a musculoskeletal (MSI) injury or sudden collapse. Other reasons for the requesting of an incident report were “galloped on (contact with another horse)” (6.2%), pulled up (16.92%) and those coded as miscellaneous (35.4%). Less than half of the incident reports (38.46%; 0.91/1000 starts, 95% CI = 0.84–0.99) had no observable abnormalities detected in the clinical findings (NOAD). 

The reporting rates of clinical observations across all incident, and non-incident stipendiary reports are presented in Table 2. Overall, integument lacerations and abrasions accounted for the largest number of incident cases (32.65%), with the remaining categories representing less than 5% of cases each and with no difference in the rates per 1000 starts. 

#### 3.2.2. Non-Incident Reports

**Figure 1:**
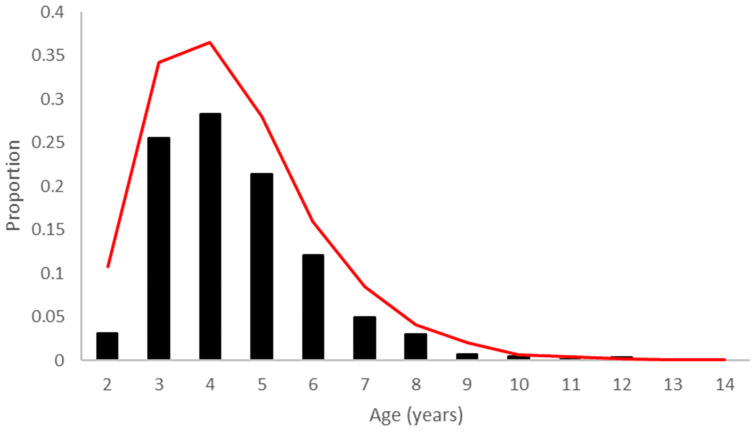
Proportion of participants (red) and the proportion of poor performance reports (black) in each age group.

There were 871 stipendiary reports that were classified as non-incident and were not associated with a race incident. The major reason for the request of a non-incident report (70.3%, 611/871) was for the routine post-race screening of horses or screening of horses performing below expectations (11.2/1000 starts, 95% CI = 10.32–12.01). Stewards were responsible for the requesting most of the poor performance examinations (95% 582/612). The distribution of age across all poor performers is presented in Figure 1 and reflected the underlying population distribution (*p* = 0.012). Over the two seasons, 87.3% (533/611) of poor performers were in the ages between 3 and 6 years old. There was a maximum of two reports for poor performance per horse in a season, with 24 horses having two reports each season. There was an association of field size with non-incident reports such that a non-incident report was 2.1 (95% CI = 1.73–2.59) times more likely to be requested in a race with 9 or more participants than in a race with 8 or less participants (*p* < 0.001).

The largest category for clinical finding of a poor performance exam was NOAD during veterinary examination (67.1%, 410/611). A small proportion of clinical findings was classified as poor recovery (6.8%), Arrhythmia (4.6%), respiratory issues (2.87%), and Lacerations/abrasions (2.64%). Musculoskeletal injuries among poor performers (1.61%) were coded as either tendon or ligament strain and myositis. 

The largest category of findings with the non-incident reports was NOAD (53% of all non-incident reports). The main clinical findings were integument lacerations and abrasions (10.2% of non-incident reports), followed by poor recovery (9.30%), arrhythmia (5.05%) and respiratory issues (4.48%). 

## 4. Discussion

The distribution of race type reported was similar to previous reports and reflects the large focus of the New Zealand harness racing industry on pacing rather than trotting races. In contrast to racing in the USA and other jurisdictions with an emphasis on racing over a mile (1609 m), many of the races in New Zealand were at a distance greater than 2000 m. Racing over these longer-distance races was reflected in the lower mile rate for both trotters and pacers [6]. The participation structure of harness racing in New Zealand has a large proportion of breeder-owner-trainers and this was reflected in the older age profile of the horses racing in New Zealand [5], which was similar to a report based on the New South Wales (Australia) harness racing population [7]. There was a reduction in the median number of horses racing and the number of starters compared to the previously published literature reflecting the reported contraction in the New Zealand racing industry [6,12].

Racing was concentrated on all-weather surfaces, around a few major venues or regions, predominantly in the South Island. This distribution reflects the underlying breeding and participation base for harness racing in New Zealand [13]. The distribution of the stewards reporting reflected this underlying distribution of harness racing in New Zealand. This in association with the relatively high level of screening (non-incident reports) indicates that the reporting should provide a good reflection of the underlying incidents and injuries observed with harness racing. The data used were collected retrospectively and cross validated with the published steward’s reports. At the time of data collection, reports were completed by officials using a *pro forma* sheet. The use of a *pro forma* reporting sheet provided some consistency in the type and level of reporting. However, despite this, there were still some limitations in ability to precisely identify the anatomical site affected and occasional inconsistency in descriptors used. During the 2018/19 racing season, an online reporting system was implemented based on the *pro forma* sheet. This online system will provide data that reflect the data previously captured using the paper-based system and the internal controls within the online system should ensure consistency in terms used with recording.

The incidence of musculoskeletal fracture during both observed seasons was lower than the incidence reported in New Zealand Thoroughbred racing (0.48 per 1000 starts) using a similar reporting framework [11]. Fractures were recorded in both incident and non-incident reports, and this reflects how an incident and non-incident report was defined. A steward may class a report with a musculoskeletal fracture as being a non-incident if there was no “event” such as a horse falling or colliding with another horse. It is also possible that some non-displaced fractures are only identifiable, because a horse performed below expectations or presented with a lameness once the horse had cooled down post racing. Within the stipendiary stewards’ reports, the description of the fractures were based on broad anatomical locations with seven of the nine fractures involving the distal limb, and predominately the first phalanx and the metacarpophalangeal joint. Fractures at these sites are typically attributed to accumulated cyclic load [14,15]. The difference in the reported fracture incidence between Thoroughbred racing and harness racing highlights the differences in the pattern and magnitude of load accumulated and the intensity of exercise between codes during both training and racing [1]. The harness racing horses had a median of 7 race starts per season over 2200 m, which was greater than the number of starts and race distance (5 starts/year, 1400 m) reported in the New Zealand Thoroughbred racing industry [13,16]. While harness racing horses in New Zealand typically acquire a greater number of load cycles during training and racing [5,17], the nature and magnitude of the load on the distal limb, and the first phalanx and metacarpophalangeal joint specifically, is less for the trotting/pacing compared to galloping [18]. The lower strain per load cycle with harness racing (trotting and pacing gait) may account for the reduced incidence as fatigue life decreases exponentially with increasing strain [19].

The rate of reporting for lacerations was moderately lower than thoroughbred racing, but similar to data reported for harness racing in Australia, which operates under similar rules and conditions to those in New Zealand [7]. The relatively higher frequency of reporting of lacerations/abrasions compared to other categories in part reflects the gait of harness racing (pace and trot) and the relatively higher risk of interference (lower limb being struck by the contralateral limb) [20]. The reporting of lameness was lower than values from Australia using a similar reporting process and the rate of reporting lameness from non-incidents was four-fold greater than with an incident report [7]. These data possibly reflect the consistent reporting that much poor performance can be associated with MSI, and that many low-grade injuries or lameness only become obvious once the horse has cooled down post-race. There was also a low rate of reporting of epistaxis (bleeders) in the dataset compared to an Australian study [7]. This could be explained by the relatively long stand down periods associated with horses that present with epistaxis on race day in New Zealand and hence a possible hesitancy of trainers to present a horse for racing if they suspect it may have an episode of epistaxis [10]. 

There was no association of track surface with the rate of incident reporting, despite the anecdotal observation that the lower-grade racing was conducted on grass surfaces rather than on all-weather. As most of the racing was conducted on all-weather tracks there may have been significant heterogeneity in the class of racing on all-weather surfaces that prevented the ability to differentiate any surface or race quality effect. In New Zealand, grass track meetings are held in the summer when weather conditions are optimal and the number of races on grass is limited to preserve the ground conditions. The type of meeting that occurs on a grass track differs to what occurs on all-weather surfaces and are part of a summer circuit where trainers take their horses on a trip to several meetings within a regional circuit. Within the current data set, it was also difficult to clearly differentiate race grade and track surface to compare the race grade effect. Harness racing data from Canada indicates that racing at higher ranking tracks was associated with a higher incidence of sudden death and accidents for horses failing to finish a race than in races at lower-ranking race tracks [4]. A similar pattern of greater MSI with higher grade racing was reported in Australian Thoroughbred racing with a higher incidence of musculoskeletal injuries reported on metropolitan racetracks compared to country track [9]. Within New Zealand, the closest analogue to metropolitan tracks for harness racing would be the all- weather surfaces, however, even on all-weather surfaces there is a large variation in race grade (quality), which may explain the lack of apparent clustering observed. 

There was no association of type of going with the rate of incident reporting. This may be explained in part by most races in New Zealand being on fast or good all-weather tracks. In New Zealand, most all-weather surfaces for harness racing consist of a predominately hardpacked lime substrate and thus there is limited variation in the material properties of fast and good all-weather surfaces and the relative speed differential between races on these going ratings over these longer distances is relatively moderate (no significant differences between categories in mile rate). There is an active program managed by Harness Racing New Zealand to ensure consistency in track surface between tracks. All harness tracks are inspected annually and concerns about track conditions are corrected prior to race day if possible. If a track is not suitable either prior to the start of the race day or during the race day, the track condition deteriorates (e.g., excessive rainfall), a meeting will be cancelled or postponed under the discretion of the stipendiary steward. By having these measures in place, injuries as a result of poor track conditions are minimized.

The number of participants in a race increased the likelihood of either an incident or non-incident report occurring. Increases in participants increases the likelihood of accidents such as horses colliding with each other or being galloped on. Similar results are reported in Thoroughbred racing where race-level factors for injuries included the type of race track, the track conditions, race distance, and field size [21]. However, the increase in the number of non-incident reports with the number of participants may reflect stewards inadvertently performing routine checks relative to the starters in the race. 

Most of the requests for a steward report were due to poor performance and the pattern of non-incident reporting appeared to be routine screening of race participants to maintain racing integrity and horse welfare. The selection of the horses for routine screening is often based on horses that had a performance in the race that was lower than the horse’s previous race, or lower than expectations as reflected by the odds at the tote (reflecting the amount of money placed/gambled on the horse via the official betting agency). Requests for examination based on the category poor performance reflected the distribution of the underlying starter population with respect to age and sex of the horse. The finding of the major category for poor performance being NOAD reflects this observation that much of these examinations were part of the routine screening process and a low frequency of undetected musculoskeletal injuries not associated with an incident during the racing event.

The primary issues identified with the social license to operate with horse racing appear to focus on the concept of injury and risk of injury to the equine participants [8]. Routine screening data, such as stewards’ reports provides metrics for industry performance. The level of stewards reporting during harness racing in New Zealand indicates that these data are representative of the industry and provides robust metrics of the industry’s performance. The low incidence of significant clinical findings from this high level of reporting and screening indicates that harness racing in New Zealand is meeting its duty of care to the horses racing in it and the primary issues associated with the social license to operate with horse racing. 

## 5. Conclusions

There was a robust level of reporting within the harness racing industry during the two seasons examined. The occurrence of non-incident and incident reports was limited emphasizing the smaller risk in New Zealand harness racing compared to Thoroughbred racing. The low fracture rate reported reflects anecdotal reporting of lower rates in harness racing compared to flat racing thoroughbreds and can be described by the different racing and training associated bone strain and fracture risk between the codes. The high level of reporting by the stewards reflects the role stewards have in maintaining of racing integrity. 

## Figures and Tables

**Table 1:** Population distribution of horses participating in the 2015/2016 and 2016/2017 New Zealand harness racing season.

	Horses per Season, and Starters, by Age Category			Sex Descriptor by Age Category (Number of Horses)					
Age (Year)	2016/17	2017/18	Starters	Colts	Fillies	Geldings	Entires/ Stallions	Mares	Rig
2	225	230	1536	59	206	188			2
3	713	729	10,025	90	670	680			2
4	790	747	14,831			797	46	691	
5	622	560	12,577			671	22	489	
6	339	331	7646			400	11	259	
7	186	172	4510			230	5	123	
8	88	85	2047			122	3	48	
9	41	43	936			62	1	21	
10	15	10	223			19		6	
11	10	7	201			13		4	
12	4	6	108			9		1	
13	1	2	45			3			
14	-	1	17			1			

**Table 2:** Frequency of clinical findings in incident (*n* = 130) and non-incident (*n* = 871) stipendiary reports over 2 complete harness racing seasons. Data are reported as frequency per 1000 starts (with 95% confidence intervals) and the percentage of clinical findings within the respective report type (incident or non-incident).

	Incident Reports		Non-Incident Reports	
Description	Frequency per 1000 Starts	%	Frequency per 1000 Starts	%
Arrhythmia	0.09 (−0.16–0.34)	3.85	0.80 (0.69–0.92)	5.05
Cardiac failure	0.02 (−0.24–0.28)	0.77		
Laceration/abrasion	0.88 (0.78–0.97)	36.92	1.63 (1.42–1.84)	10.22
Lame	0.09 (−0.16–0.34)	3.85	0.40 (0.20–0.61)	2.53
MSI Fracture	0.09 (−0.16–0.34)	3.85	0.07 (−0.18–0.33)	0.46
Other MSI issues	0.09 (−0.16–0.34)	3.85	0.57 (0.39–0.74)	3.56
Unknown MS pain	0.07 (−0.18–0.33)	3.08	0.44 (0.24–0.64)	2.76
No observable abnormalities detected (NOAD)	0.77 (0.0.64–0.90)	32.31	8.39 (7.67–9.11)	52.70
Poor recovery	0.11 (−0.14–0.36)	4.62	1.48 (1.30–1.66)	9.30
Respiratory issues	0.09 (−0.16–0.34)	3.85	0.71 (0.57–0.85)	4.48
Previous injury			0.24 (0.01–0.47)	1.49
Bleeders (epistaxis)			0.26 (0.03–0.48)	1.61
Unknown			0.33 (0.11–0.55)	2.07
Miscellaneous	0.07 (−0.18–0.33)	3.08	0.60 (0.44–0.77)	3.79

## Data Availability

Source data are publicly available via respective websites, collated extracts are the property of respective bodies.
